# Connector blood leakage suggesting pulmonary artery catheter damage

**DOI:** 10.1002/anr3.70022

**Published:** 2025-07-22

**Authors:** T. Ishii, H. Miyoshi, H. Sato, H. Yokomi, T. Takasaki, S. Takahashi, Y. M. Tsutsumi

**Affiliations:** ^1^ Department of Anaesthesiology and Critical Care Hiroshima University Hiroshima Japan; ^2^ Department of Cardiovascular Surgery Hiroshima University Hiroshima Japan

**Keywords:** cardiac surgical procedures, catheterisation, Swan‐Ganz, intra‐operative complications

Catheter entrapment is a rare complication of pulmonary artery catheter (PAC) insertion [1, 2].

A 57‐year‐old man underwent ascending aortic replacement. A PAC was inserted via the right internal jugular vein without difficulty under continuous transoesophageal echocardiographic guidance, which confirmed its appropriate placement in the right pulmonary artery. As this was his second cardiac surgery, dense adhesions were present. During adhesiolysis, an injury occurred at the base of the pulmonary artery, which was successfully repaired using felt‐reinforced sutures.

**Figure 1:**
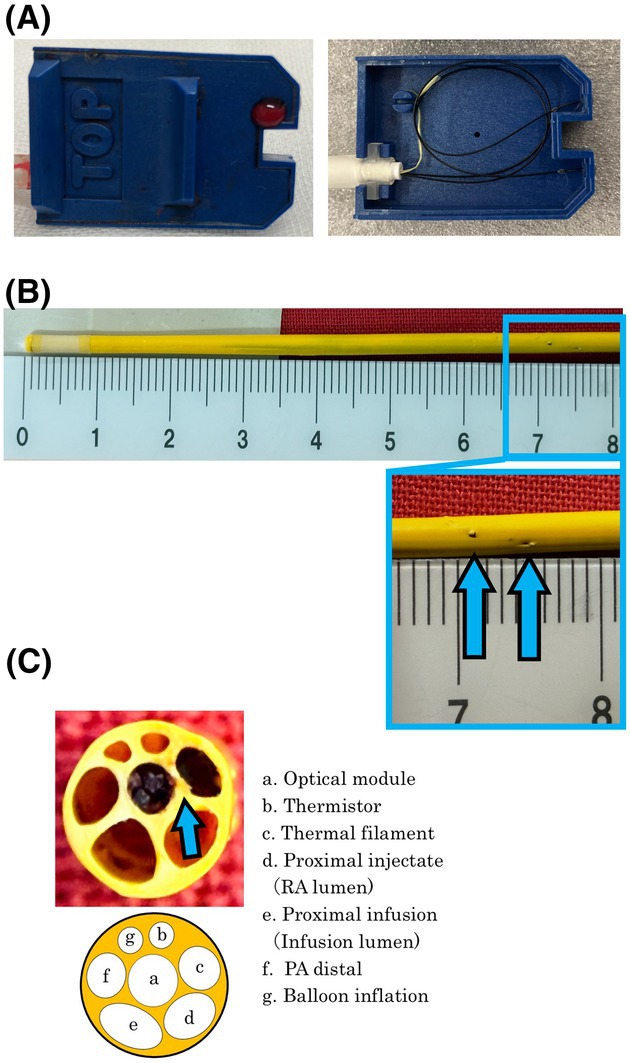
(A) Left – blood leakage observed around the optical module connector; right – internal structure of the device (normal); (B) the removed catheter was sutured at 7.1 cm and 7.5 cm (arrows) from the catheter tip; (C) cross‐sectional view of the catheter at 7.1 cm with damage to the walls of the optical module (arrow). PA, pulmonary artery; RA, right atrium.

Fresh blood was observed around the optical module connector upon arrival to the intensive care unit, though the significance of this was unknown (Fig. 1A). Pulmonary artery pressures, mixed venous oxygen saturation and cardiac output measurements were unaffected and clinically appropriate.

On postoperative day 1, PAC removal was attempted but resistance was encountered after withdrawing approximately 5 cm. Fluoroscopy revealed that the catheter tip was fixed. During resternotomy, we found the PAC had been inadvertently sutured to the pulmonary artery. The catheter was carefully released and removed with cardiac bypass support. Examination revealed suture punctures through the optical module lumen, resulting in blood leakage without affecting monitored values (Fig. 1B and C). The patient recovered without further complications.

Blood leakage from the optical module connector may indicate catheter damage or entrapment, even in the absence of abnormal monitoring data, and should prompt further evaluation.
